# The Association of Radiation Dose With Overall Survival for Patients Treated With Prostate Stereotactic Body Radiation Therapy

**DOI:** 10.7759/cureus.34351

**Published:** 2023-01-29

**Authors:** Michael R Waters, Neal Andruska, Benjamin W Fischer-Valuck, Temitope Agabalogun, Randall J Brenneman, Hiram Gay, Jeff M Michalski, Brian Baumann

**Affiliations:** 1 Radiation Oncology, Washington University School of Medicine, St. Louis, USA; 2 Radiation Oncology, Emory University School of Medicine, Atlanta, USA; 3 Radiation Oncology, Washington University School of Medicine, St. Loui, USA

**Keywords:** intermediate and high risk prostate cancer, national cancer database and seer analyses, general radiation oncology, stereotactic body radiotherapies, prostate hypofractionation

## Abstract

Introduction

Stereotactic body radiation therapy (SBRT) for prostate adenocarcinoma (PCa) has demonstrated excellent biochemical recurrence-free survival, with studies showing improved BRFS with higher-dose SBRT. However, current studies have been underpowered to evaluate the relationship of SBRT dose to overall survival (OS). In this retrospective study using the National Cancer Database (NCDB), we hypothesize that, given the low alpha/beta ratio of PCa, a relatively small increase in the dose-per-fraction would be associated with improved survival outcomes for intermediate-risk PCa (IR-PCa) comparing 36.25 Gy/5 fx [biologically equivalent dose (BEDα/β = 1.5 = 211.46 Gy vs. 35 Gy (BED1.5 = 198.33 Gy)].

Materials and methods

We queried records from the NCDB from 2005 to 2015 for men receiving prostate SBRT for IR-PCa (n=2673). 82% were treated using either 35 Gy/5 fx or 36.25 Gy/5 fx. We compared OS in men receiving 35 Gy versus 36.25 Gy. Inverse probability of treatment weighting (IPTW) was used to adjust for covariable imbalances. Unweighted- and weighted-multivariable analysis (MVA) using Cox regression was used to compare OS hazard ratios, accounting for age, race, Charlson-Deyo comorbidity score, treatment facility type, prostate-specific antigen (PSA), clinical T-stage, Gleason Score, and use of androgen deprivation therapy (ADT). Kaplan-Meier analysis was performed.

Results

Seven hundred and eighty men (35%) were treated with 35 Gy/5 fx and 1434 men (65%) were treated with 36.25 Gy/5 fx (n=2214). Compared to 35 Gy, treatment with 36.25 Gy was associated with significantly improved OS (hazard ratio [HR]: 0.61 [95% CI: 0.43-0.89], *P=*0*.*009) on MVA. On Kaplan-Meier analysis, 36.25 Gy was associated with improved survival (p=0.034), with a five-year OS of 92% and 88%, respectively.

Conclusions

In a multi-institutional retrospective database of 2,214 IR patients treated with prostate SBRT, a prescription dose of 36.25 Gy/5 fx was associated with improved OS vs. 35 Gy/5 fx. Results are hypothesis-generating but do lend support to the current National Comprehensive Cancer Network (NCCN) guidelines that the minimum recommended dose for prostate SBRT is 36.25 Gy/5 fx.

## Introduction

Stereotactic body radiation therapy (SBRT) to the prostate has recently emerged as an alternative treatment modality for intermediate-risk prostate cancer (IR-PCa). Studies evaluating the utility of SBRT in prostate cancer (PCa) have reported excellent biochemical-free survival (BRFS) [1-3], though limited follow-up from randomized trials has slowed the widespread adoption of prostate SBRT. Further, the optimal dose for prostate SBRT has not been determined.

Several large randomized trials have confirmed that dose escalation results in improved relapse-free survival and reduced distant metastasis rates in patients with PCa treated with standard fractionation [4,5]. Similarly, in a large multi-institutional study of SBRT in low and intermediate-risk PCa, SBRT dose escalation to 40 Gy in five fractions was associated with improved biochemical recurrence-free survival [6]. However, to date, studies have been underpowered to evaluate the relationship of SBRT dose to distant metastasis-free survival or overall survival (OS).

Importantly, the relative biological effect (RBE) of SBRT dose escalation is magnified in PCa compared to other cancers given its low alpha/beta (α/β) ratio, which has been estimated at 1-2 [7-9]. Thus, a relatively small increase in total dose, in the context of hypofractionation, can lead to a dramatic increase in the biologically equivalent dose (BED) in PCa [10]. To evaluate the clinical utility of BED escalation with hypofractionated treatment regimens in PCa, clinical trials are underway [11].

Current National Comprehensive Cancer Network (NCCN) guidelines recommend at least 36.25 Gy/5 fx, but former NCCN guidelines suggested lower SBRT doses (e.g., 35 Gy/5) were acceptable. The impact of a lower dose of prostate SBRT (35 Gy/5 fx) on survival outcomes has not been assessed in a large cohort. In this retrospective study, we evaluated the effect of dose escalation on overall survival by comparing the two most common SBRT dose prescriptions in the NCDB.

## Materials and methods

NCDB patient cohort

Patients diagnosed with biopsy-proven PCa and treated with SBRT between 2005 and 2015 were queried by the National Cancer Database (NCDB). This cohort was filtered for patients meeting NCCN criteria for IR-PCa [12]. Of these patients, 82% received either 35 Gy/5 fx or 36.25 Gy/5 fx and were included in the analysis. Both of these prescriptions were consistent with NCCN guidelines during the time of the study.

Statistical analysis

Overall survival (OS) was the primary endpoint. Chi-square and Student's t-tests were used to detect significant differences among categorical and continuous variables, respectively. The Kaplan-Meier method was used to estimate OS, and log-rank tests were used to compare different treatment arms. Multivariable OS hazard ratios were estimated using Cox regression analysis. Overall model significance was assessed using the Wald statistic.

The inverse propensity of treatment weighting (IPTW) was used to adjust for covariable imbalance. Inverse probability of treatment weighting has been used in observational studies to reduce selection bias. Inverse-probability weighting removes confounding by creating a pseudo-population in which the treatment is independent of the measured cofounders through the weighting of observations. Survival was estimated using a multivariable linear regression model to generate propensity scores. Covariables selected a priori for analysis included the age, race, Charlson-Deyo comorbidity index, treatment at an academic center, pre-treatment prostate-specific antigen (PSA), clinical T-stage, Gleason score, and addition of androgen deprivation therapy (ADT).

Unstabilized inverse propensity weights were generated, with truncation of the most extreme weights as previously described (α = 0.0001) [13], and a pseudo-sample population in which measured baseline covariables were balanced between treatment groups was generated. Acceptable covariable balance among treatment groups was verified using the standardized mean difference, with a standardized mean difference less than 0.1 (10%) considered negligible [14].

Multivariable analysis (MVA) was performed with weights applied to the time-dependent Cox proportional hazard model to compare the effects of 35 Gy vs. 36.25 Gy. IPTW Kaplan-Meier curves were generated as the weighted product limit estimator. Additional details of this approach have been published previously [13-15]. All tests were two-sided. P<0.05 were considered statistically significant.

## Results

This study included 2214 men with IR-PCa treated with SBRT either to (i) 35 Gy/5 fractions (n=780), or (ii) 36.25 Gy/5 fractions (n=1434) corresponding to a BED (α/β=1.5) of 198.33 Gy and 211.46 Gy, respectively. The median follow-up time was 35.5 months, with 139 deaths. The cohorts did not differ significantly on univariate comparison of age, PSA, T-stage, Gleason group, or treatment at an academic center (p > 0.05) (Table 1). Of note, the group receiving 36.25 Gy received a higher proportion of ADT on Chi-Square analysis (12% vs. 5%, p = 3.4 × 10^−^^7^). Given this difference, ADT was incorporated into both the unweighted multivariable analysis and the IPTW-MVA.

**Table 1:** Baseline characteristics of patients treated with SBRT using a cumulative dose of 35 Gy versus 36.25 Gy. SBRT: stereotactic body radiation therapy, PSA: prostate-specific antigen, ADT: androgen deprivation therapy.

	Total	35 Gy	36.25 Gy	P-value
Total patients, n	2216	781	1435	
Follow-up, mean (SD)	40	36	42	
Age, mean (SD)	68	67	68	0.007
Race				
White	1814	626	1188	0.21
Black	318	126	192	
Other	84	29	55	
Spanish or Hispanic origin				
Non-Spanish, non-Hispanic	2125	758	1367	0.054
Spanish or Hispanic	91	23	68	
CDCI (comorbidity) score				
0	1931	663	1268	0.02
1	285	118	167	
Treatment at an academic center				
No	1039	187	852	<2.2 × 10^−^^16^
Yes	1177	594	583	
Year of diagnosis				
2004–2007	30	12	18	
2008–2010	824	262	562	0.03
2011–2015	1362	507	855	
PSA, mean (SD)	7.4	7.4	7.4	0.95
Gleason score				
3+3	228	81	147	
3+4	1327	437	890	0.02
4+3	661	263	398	
Clinical T-stage				
≤cT2a	1921	671	1250	
T2b-T2c	248	85	163	0.03
cT2, NOS	47	25	22	
ADT				
No	1994	737	1257	
Yes	222	44	178	5.8 × 10^−^^7^

An unweighted MVA was employed to assess the effect of an increased SBRT dose on OS. On MVA analysis, treatment with 36.25 Gy was associated with significantly improved OS (hazard ratio (HR): 0.61 [95% CI: 0.43-0.89], P = 0.009) compared to 35 Gy (Table 2). Additionally, older age was also associated with the reduced OS on MVA (HR: 1.05 [1.02-1.07], P<0.001). PSA (HR: 1.03 [0.98-1.08], P=0.19) and higher T-stage (HR: 1.53 [0.97-2.39], P=.07) trended towards significance (Table 2).

**Table 2:** Unweighted multivariable analysis of overall survival for patients treated with SBRT at 35 Gy/5 fractions or 36.25 Gy/5 fractions. SBRT: stereotactic body radiation therapy, PSA: prostate-specific antigen, ADT: androgen deprivation therapy. ^‡^A CDCI score of 1 corresponds to men with a history of one of the following: myocardial infarction, congestive heart failure, peripheral vascular disease, cerebrovascular disease, dementia, chronic pulmonary disease, rheumatologic disease, peptic ulcerdisease, mild liver disease, or diabetes.

	HR [95% CI]	P-value
Age	1.04 [1.01–1.09]	0.0002
Race		
White	1	-
Black	1.04 [0.61–1.76]	0.9
Other	0.72 [0.22–2.31]	0.58
Spanish or Hispanic origin		
Non-Spanish	1	-
Non-Hispanic Spanish or Hispanic	1.30 [0.62–2.71]	0.49
CDCI (comorbidity) score		
0	1	-
1^‡^	1.26 [0.80–1.99]	0.32
Treatment at an academic center		
No	1	-
Yes	0.70 [0.49–1.00]	0.056
Year of diagnosis		
2004–2007	1	-
2008–2010	1.96 [0.85–4.53]	0.12
2011–2015	2.40 [0.96–6.06]	0.06
PSA	1.03 [0.98–1.08]	0.19
Gleason score		
3+3	1	-
3+4	1.83 [0.89–3.74]	0.1
4+3	1.95 [0.92–4.13]	0.08
Clinical T-stage		
≤cT2a	1	-
T2b-T2c	1.53 [0.97–2.39]	0.07
cT2, NOS	1.40 [0.43–4.50]	0.57
ADT		
No	1	-
Yes	1.57 [0.97–2.53]	0.07
SBRT dose		
35 Gy/5	1	-
36.25 Gy/5	0.60 [0.42–0.87]	0.007

**Figure 1:**
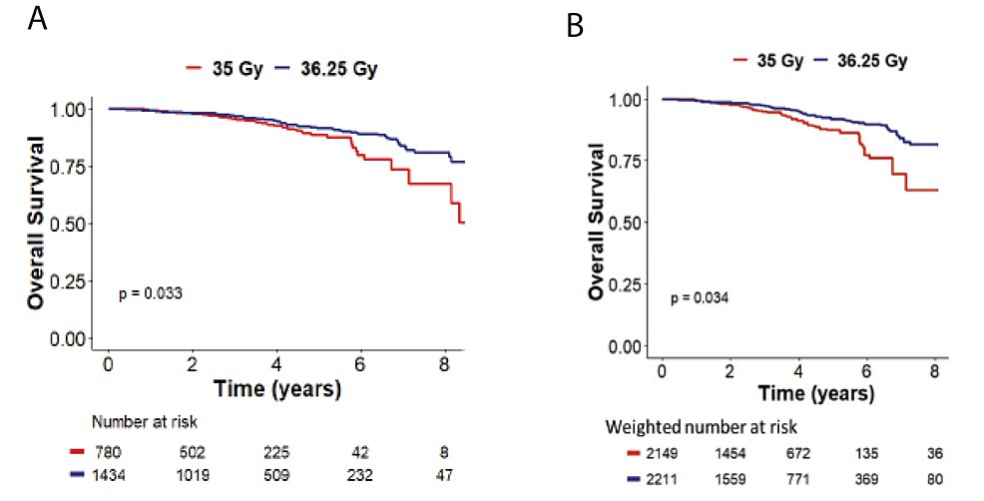
(A) Unweighted versus (B) inverse probability of treatment weighting-adjusted Kaplan-Meier curves stratified by SBRT dose (35 Gy vs. 36.25 Gy). SBRT: stereotactic body radiation therapy.

In addition to unweighted MVA, IPTW-MVA was used to balance co-variables with potential influence on both treatment allocation and outcomes. Weight-adjusted Cox regression was used to determine the average treatment effect of an increased SBRT dose on groups with balanced confounders (Table 3). IPTW analysis confirmed that an SBRT dose of 36.25 Gy was significantly associated with improved OS compared to 35 Gy (p=0.0091) (Table 3). The patient’s race, treatment at an academic center, PSA, T-stage, Gleason score, and treatment with androgen deprivation therapy were not associated with survival (Table 3). Lastly, using Kaplan-Meier techniques, treatment with 36.25 Gy was associated with improved OS vs. 35 Gy (p=0.034). Five-year overall survival for 35 Gy and 36.25 Gy was 88% and 92%, respectively (Figure 1).

**Table 3:** MVA showing the average treatment effect of SBRT to a dose of 35 Gy versus 36.25 Gy using IPTW-weighted OS hazard ratios. SBRT: stereotactic body radiation therapy, MVA: multivariable analysis, IPTW: inverse propensity of treatment weighting, OS: overall survival, PSA: prostate-specific antigen, ADT: androgen deprivation therapy. ^‡^A CDCI score of 1 corresponds to men with a history of one of the following: myocardial infarction, congestive heart failure, peripheral vascular disease, cerebrovascular disease, dementia, chronic pulmonary disease, rheumatologic disease, peptic ulcerdisease, mild liver disease, or diabetes.

	HR [95% CI]	P-value
Age	1.05 [1.02–1.09]	0.006
Race		
White	1	-
Black	0.85 [0.48–1.50]	0.57
Other	1.01 [0.40–2.58]	0.98
Spanish or Hispanic origin		
Non-Spanish	1	-
Non-Hispanic Spanish or Hispanic	0.76 [0.32–1.77]	0.52
CDCI (comorbidity) score		
0	1	-
1^‡^	1.17 [0.71–1.92]	0.55
Treatment at an academic center		
No	1	-
Yes	0.75 [0.51–1.10]	0.15
Year of diagnosis		
2004–2007	1	-
2008–2010	1.24 [0.50–3.10]	0.64
2011–2015	2.08 [0.70–6.13]	0.19
PSA	0.99 [0.94–1.05]	0.79
Gleason score		
3+3	1	-
3+4	1.43 [0.67–3.06]	0.29
4+3	1.57 [0.69–3.58]	0.13
Clinical T-stage		
≤cT2a	1	-
T2b-T2c	1.35 [0.83–2.20]	0.23
cT2, NOS	1.28 [0.48–3.38]	0.62
ADT		
No	1	-
Yes	1.50 [0.84–2.65]	0.17
SBRT dose		
35 Gy/5	1	-
36.25 Gy/5	0.61 [0.42–0.88]	0.009

## Discussion

In a retrospective study of 2214 men with IR-PCa cancer treated definitively with prostate-directed SBRT, patients treated to 36.25 Gy had excellent survival outcomes, with an association with improved OS in patients treated with 36.25 Gy/5 fx compared to 35 Gy/5 fx. These findings confirm current NCCN recommendations that patients should be treated to a dose of ≥36.25 Gy when using SBRT.

Our findings align with prior studies, which demonstrate that prostate-directed SBRT results in excellent oncologic outcomes in IR-PCa. A large retrospective meta-analysis of prostate SBRT patients with low, intermediate, and high-risk diseases reported a seven-year biochemical relapse-free survival rate of 93.7% [7,16,17]. Further, the American Society for Radiation Oncologists (ASTRO), American Society of Clinical Oncology (ASCO), and NCCN guidelines indicate that prostate SBRT is an acceptable treatment strategy for low and intermediate-risk prostate cancer [12,17].

While prior studies have reported improved biochemical recurrence-free survival with dose-escalated SBRT versus lower dose SBRT [6], these studies were not powered to detect a potential improvement in overall survival or distant metastasis-free survival, which can be used as a surrogate endpoint for overall survival in prostate cancer. Our study adds to the current literature by comparing two common SBRT dose prescriptions with a sufficiently large sample size to evaluate for differences in survival outcomes between the two dose prescriptions. The improvement in OS associated with 36.25 Gy (BED 211.46 Gy, α/β=1.5) versus 35 Gy (BED 198.33 Gy) persisted with the inclusion of potential cofounders in the model, including age, race, treatment at an academic center, comorbidity index, T stage, PSA, Gleason score, and addition of hormone therapy. While the difference in absolute dose appears relatively modest, we hypothesize that the observed association with improved survival is due to the substantial increase in BED going from 35 Gy/5 fx to 36.25 Gy/5 fx [a BED increase of 13 Gy (α/β=1.5) or an increase of 16 Gy (α/β=1.2)]. It should be noted that NRG 0126, which compared 70 Gy vs. 79.2 Gy in 1.8 Gy/fraction in a similar intermediate-risk patient population (n=1532), reported a significant reduction in distant metastasis with dose-escalated treatment with a relatively comparable difference in BED between the two arms. It is therefore possible that in a larger cohort of patients treated with SBRT, the benefit of reduced distant metastasis with dose escalation seen in RTOG 0126 may translate to a small but statistically significant improvement in overall survival. Our findings support the current NCCN recommendation that prostate SBRT should be delivered at a dose of ≥36.25 Gy and suggest that 35 Gy may be suboptimal.

This study has several important limitations. The NCDB only records all-cause mortality and does not record recurrence or cancer-specific mortality, which precludes our ability to perform disease-free survival or prostate cancer-specific mortality analysis, a key limitation of the study. In addition, the NCDB provides the prescription dose but does not provide data on prescription coverage for the target, which could have an impact on oncologic outcomes. While we adjusted for measured confounders in the inverse propensity weighting model, such as academic versus non-academic treatment center, age, Gleason score, PSA, comorbidity score, and use of ADT, we were not able to account for unmeasured confounders, which could play a role in whether patients were treated with 36.25 Gy or 35 Gy. During the study period from 2005 to 2015, both 36.25 Gy and 35 Gy were consistent with NCCN guidelines, with many centers standardizing a particular prescription dose for their SBRT patients. While the use of a standardized SBRT dose may reduce some patient-level selection bias for patients treated by the same physician or at the same institution, selection bias in this non-randomized cohort remains an important limitation. Over the study period, stage migration of Gleason grading could potentially impact the results. Ascertainment bias is also a potential concern in these large retrospective databases. The study also does not report toxicity data, which is not available in the NCDB. Higher-dose SBRT, while associated with improved survival in our analysis, is also likely to be associated with a higher risk of adverse events. Fortunately, rates of late-grade ≥3 GI and GU adverse events with SBRT were 2.0% and 1.1%, respectively, in a large pooled analysis of SBRT patients [16].

## Conclusions

In this retrospective study using the NCDB, we hypothesized that given the low alpha/beta ratio of PCa, a relatively small increase in the dose-per-fraction would be associated with improved survival outcomes for IR- PCa comparing 36.25 Gy/5 fx [(BED α/β=1.5=211.46 Gy vs. 35 Gy (BED1.5=198.33 Gy)]. In a large, multi-institutional retrospective database of 2214 IR prostate cancer patients treated with SBRT, a prescription dose of 36.25 Gy in 5 fractions was associated with improved OS vs. 35 Gy in 5 fractions. These results are hypothesis-generating, but they do support current NCCN guidelines, in which the minimum recommended dose for prostate SBRT is ≥36.25 Gy/5 fx.
